# Comparison of Crystal Field Dependent and Independent Methods to Analyse Lanthanide Induced NMR Shifts in Axially Symmetric Complexes. Part II: Systems with a C_4_ Symmetry Axis

**DOI:** 10.1155/S1565363303000013

**Published:** 2003

**Authors:** Carlos F. G. C. Geraldes, Shanrong Zhang, A. Dean Sherry

**Affiliations:** 1 Department of Biochemistry and Center of Neurosciences University of Coimbra P.O. Box 3126 Coimbra 3001-401 Portugal; 2 Department of Chemistry The University of Texas at Dallas P. O. BOX 830688 Richardson TX 75083-0688 USA; 3 The Mary Nell and Ralph B. Rogers Magnetic Resonance Center Department of Radiology University of Texas Southwestern Medical Center 5801 Forest Park Rd. Dallas TX 75235-9085 USA

## Abstract

Analysis of the LIS data for several series of Ln^3+^ complexes of C_4_ symmetry in terms of structural changes, crystal-field effects and/or variation of hyperfine constants along the lanthanide series was undertaken using a combination of the two-nuclei and three-nuclei techniques together with the classical onenucleus technique. Isostructurality of whole series of complexes, with changes of the F_i_, and B_0_^2^ parameters, was clearly defined for the complexes of L by the combination of the two first methods. Small changes, involving the three F_i_, G_i_ and B_0_^2^ parameters, are observed for the series of complexes of L-L4, using the three data plotting methods. Some of the plots according to the two- and three-nuclei methods are accidentally linear, without necessarily implying isostructurality of the complexes, as they involve parameters, which may be insensitive to any small structural changes occurring in these systems. These parameter variations could result from a magnification, by the present graphical analysis, of the breaks expected from the gradual structural changes along the series due to the lanthanide contraction. The α and β parameters of the three-nuclei method are not diagnostic of the type of structures the complexes have in solution, due to their very indirect dependence on the geometric factors.


### Comparison of Crystal Field Dependent and Independent Methods to Analyse Lanthanide Induced NMR Shifts in Axially Symmetric Complexes. Part II: Systems with a $C_4$ Symmetry Axis

Carlos F. G. C. Geraldes[a,*], Shanrong Zhang[b] and A. Dean Sherry[b,c]

[a]*Department of Biochemistry and Center of Neurosciences, University of Coimbra, P.O. Box 3126, 3001-401 Coimbra, Portugal*
[b]*Department of Chemistry, The University of Texas at Dallas, P. O. BOX 830688, Richardson, TX 75083-0688*
[c]*The Mary Nell and Ralph B. Rogers Magnetic Resonance Center, Department of Radiology, University of Texas Southwestern Medical Center, 5801 Forest Park Rd., Dallas, TX 75235-9085*

(Received: November 27, 2002; Accepted: January 22, 2003)

**ABSTRACT**

Analysis of the LIS data for several series of $Ln^{3+}$ complexes of $C_4$ symmetry in terms of structural changes, crystal-field effects and/or variation of hyperfine constants along the lanthanide series was undertaken using a combination of the two-nuclei and three-nuclei techniques together with the classical one-nucleus technique. Isostructurality of whole series of complexes, with changes of the $F_i$, and $B_0^2$ parameters, was clearly defined for the complexes of $L^5$ by the combination of the two first methods. Small changes, involving the three $F_i$, $G_i$ and $B_0^2$ parameters, are observed for the series of complexes of $L^1$-$L^4$, using the three data plotting methods. Some of the plots according to the two- and three-nuclei methods are accidentally linear, without necessarily implying isostructurality of the complexes, as they involve parameters, which may be insensitive to any small structural changes occurring in these systems. These parameter variations could result from a magnification, by the present graphical analysis, of the breaks expected from the gradual structural changes along the series due to the lanthanide contraction. The α and β parameters of the three-nuclei method are not diagnostic of the type of structures the complexes have in solution, due to their very indirect dependence on the geometric factors.

---

* Corresponding author: Carlos F. G. C. Geraldes
Department of Biochemistry, Faculty of Science and Technology,
University of Coimbra, P.O. Box 3126, 3001-401 Coimbra, Portugal
Phone: 00351239853608; Fax: 00351239853607; Email: geraldes@ci.uc.pt

**Keywords:** Solution Structure by NMR, Lanthanide Complexes, Lanthanide Induced Shifts

### INTRODUCTION

The binding of a ligand to a paramagnetic trivalent lanthanide metal ion, $Ln^{3+}$, generally results in large hyperfine NMR shifts (LIS) as well as nuclear relaxation enhancements (LIR) at the ligand nuclei, which have made them ideal structural probes of supramolecular complexes and proteins /1/, while $Gd^{3+}$ complexes have found very useful biomedical applications as contrast agents for Magnetic Resonance Imaging /2/. In particular, the LIS have magnitudes and signs depending critically on both the nature of the $Ln^{3+}$ ion and the location of the nucleus relative to the metal center, making them very sensitive to structural changes /1,3,4/. In fact, the observed LIS values, $\delta_{ij}^{para}$, induced by a paramagnetic $Ln^{3+}$ ion $j$ upon the NMR signal of a nucleus $i$, resulting from the coupling of the electronic magnetic moment of $j$ with the nuclear magnetic moment of $i$, has two contributions /1c,5-9/, a through-bond Fermi contact ($\delta_{ij}^{c}$) and a through-space dipolar or pseudo-contact shift ($\delta_{ij}^{d}$), which in an axially symmetric complex (with at least a $C_3$ or $C_4$ symmetry axis) can be written as /10/:

$$\delta_{ij}^{para} = \delta_{ij}^{c} + \delta_{ij}^{d} = F_i \langle S_z \rangle_j + C_j B_2^0 G_i \qquad (1)$$

where $F_i$ is proportional to the hyperfine coupling constant $A_i$ of nucleus $i$, $\langle S_z \rangle_j$ is the spin expectation value for the paramagnetic $Ln^{3+}$, $C_j$ is Bleaney's factor, a magnetic constant measuring at a given temperature the second-order magnetic axial anisotropy of the paramagnetic lanthanide $j$ (scaled to −100 for Dy), $B_2^0$ is the axial second-order crystal field parameter of the complex, and $G_i$ is the axial geometric factor of nucleus $i$, [$G_i = (3\cos^2\theta_i - 1)/r_i^3$], where $r_i$ and $\theta_i$ are the axial polar coordinates of nucleus $i$ in the principal axis ($z$-axis) of the magnetic susceptibility tensor of the complex, with the $Ln^{3+}$ ion at the origin. Because only the dipolar term contains the geometric information of interest about the lantanide complex, any quantitative structural analysis requires a reliable separation of the observed shift into the contact and dipolar terms. An empirical separation method has been proposed /11/, based on measurement of LIS data for a series of lanthanide complexes. This one-nucleus technique uses plots based on rearrangement of eq. (1) into two linear forms (eqs. (2) and (3)):

$$\frac{\delta_{ij}^{para}}{\langle S_z \rangle_j} = F_i + B_2^0 G_i \frac{C_j}{\langle S_z \rangle_j} \qquad (2)$$

$$\frac{\delta_{ij}^{para}}{C_j} = F_i \frac{\langle S_z \rangle_j}{C_j} + B_2^0 G_i \qquad (3)$$

The LIS separation is then achieved at a fixed temperature with the following assumptions: (1) the $\langle S_z \rangle_j$ and $C_j$ parameters tabulated for the $Ln^{3+}$ free ions /5,7,12/ are a valid approximation for all complexes; (2) the hyperfine coupling constants ($A_i$, and hence the $F_i$ terms) for each nucleus, and the crystal field parameter, $B_2^0$, are invariant along the lanthanide series. Often plots of the observed LIS data according to eqs. (2) and (3) are linear along the lanthanide series. Then, $F_i$ and $(B_2^0 G_i)$ can be determined by linear regression and the above assumptions are proven to be valid, in particular that the complexes are isostructural and the crystal field coefficient is invariant along the $Ln^{3+}$ series. However, breaks frequently found in such plots near the middle of the Ln series (Gd–Dy) /1c/ have in some cases been ascribed to a gradual change of the $G_i$ factors associated with the lanthanide contraction /13/, an effect which is amplified for the heavier lanthanides because of their large $C_j$ values /6/. However, in other cases such breaks have been assigned to variations of $F_i$ and/or $B_2^0$ along the Ln series /14-17/.

Combination of eq. (1) for the paramagnetic shifts ($\delta_{ij}^{para}$ and $\delta_{kj}^{para}$) of two nuclei $i$ and $k$, in the same complex, leads to the removal of the crystal field parameter $B_2^0$ in eq. (4) /14,18/:

$$\frac{\delta_{ij}^{para}}{\langle S_z \rangle_j} = (F_i - F_k R_{ik}) + R_{ik} \frac{\delta_{kj}^{para}}{\langle S_z \rangle_j} \qquad (4)$$

where $R_{ik} = G_i/G_k$, which can be used to investigate the isostructurality of complexes along the lanthanide series. In fact, since eq. (4) does not depend on the crystal field parameter, any deviation from linearity found on plots of $\delta_{ij}^{para}/\langle S_z\rangle_j$ *vs.* $\delta_{kj}^{para}/\langle S_z\rangle_j$ along the Ln series can be safely ascribed to structural changes affecting $R_{ik}$, and thus $G_i$ and $G_k$ /18-20/.

Because theoretical $\langle S_z\rangle_j$ and $C_j$ values for some of the lanthanide ions, in particular for Sm and Eu /5,7,21/, have anomalous temperature dependencies, there is a lack of reliable theoretical $\langle S_z\rangle$ and $C_j$ values for either abnormally high or low temperatures. Thus, although these parameters are relatively independent from crystal field effects around room temperature, it is desirable to analyze LIS data and to test for isostructurality of series of lanthanide complexes without recourse to the use of both $B_2^0$ of the complexes and the theoretical $\langle S_z\rangle_j$ and $C_j$ free-ion parameters. This can be achieved with the three-nuclei method /22/, based on eq. (5) for three different nuclei $i$, $k$, $l$ in the same lanthanide complex:

$$\frac{\delta_{ij}^{para}}{\delta_{kj}^{para}} = \alpha \frac{\delta_{lj}^{para}}{\delta_{kj}^{para}} + \beta \qquad (5)$$

where $\alpha = [(F_i/F_k) - R_{ik})]/[(F_l/F_k) - R_{lk})]$ and $\beta = [(F_l/F_k) R_{ik} - (F_i/F_k) R_{lk})]/[(F_l/F_k) - R_{lk})]$. Plots of $(\delta_{ij}^{para}/\delta_{kj}^{para})$ *vs.* $(\delta_{lj}^{para}/\delta_{kj}^{para})$ for a series of lanthanide complexes are linear, with slope $\alpha$ and intercept $\beta$, provided that the complexes are isostructural and the hyperfine coupling constants are invariant along the lanthanide series. As it is solely based on experimental LIS data, this method can be applied to any ligand for which the LIS data are available for at least three nuclei, over a very wide range of temperatures.

Complementary structural information on the complexes is provided by the experimental relative Ln-$H_j$ distance values ($r_{Hj}$), obtained from the paramagnetic contribution to the proton spin-lattice ($T_1$) and spin-spin

($T_2$) relaxation times, by eq. (6) /1/:

$$r_{Hj} / r_{Hk} = (T_{i,Hk} / T_{i,Hj})^{1/6} \qquad i = 1,2 \tag{6}$$

The relative distances obtained by eq. (6) should be independent of the electronic spin relaxation time, rotational correlation time and magnetic moment of each individual complex.

The combined use of the above two-nuclei and three-nuclei techniques together with the classical one-nucleus technique, according to eqs. (1)-(5), to study the LIS values for a series of lanthanide complexes, is particularly powerful in assigning eventual structural changes, crystal-field effects and/or variation of hyperfine constants along the lanthanide series /19,22-26/. A critical study of the results of this approach to the analysis of LIS data of a series $Ln^{3+}$ complexes of three-fold symmetry (at least a $C_3$ axis) in terms of structural changes, crystal-field effects and/or variation of hyperfine constants along the lanthanide series, has recently been undertaken /26,27/. We now extend this analysis to the LIS data available for complexes of four-fold symmetry (at least a $C_4$ axis) of linear an macrocyclic ligands in different stoichiometries, solvents and temperatures (see Fig. 1).

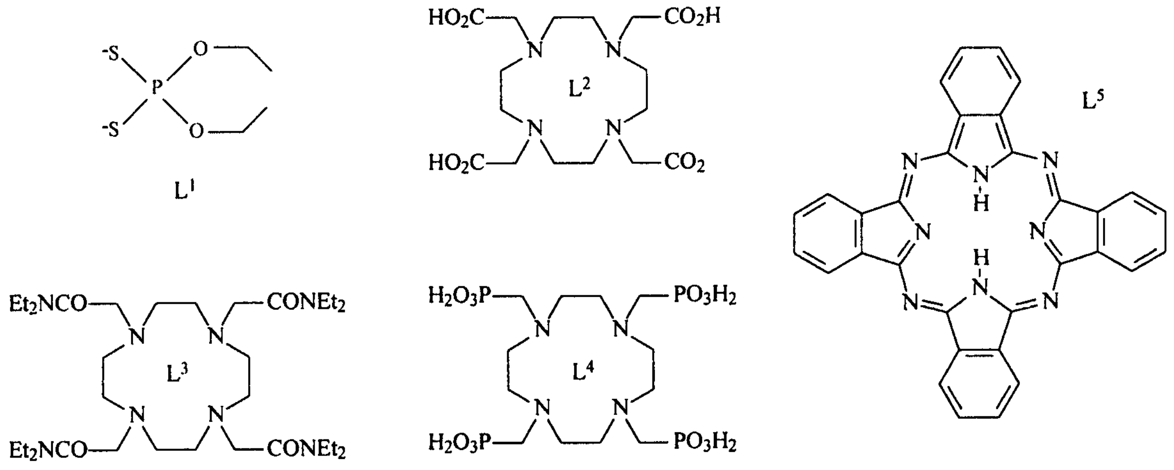

**Fig. 1:** Chemical structure of the ligands cited in this work.

### RESULTS AND DISCUSSION

### $[Ln(R_2PS_2)_4]^-$ ($L^1 = R_2PS_2^-$ = dithiomethylphosphinate and dithiophosphate derivatives).

A large number of lanthanide complexes of the $[Ln(R_2PS_2)_4]^-$ type has been isolated and characterized in the solid state by X-ray crystallography, where the ligand of general formula $R_2PS_2^-$, with two sulphur donor atoms, is a dithiophosphinate (eg. R = Me) or a dithiophosphonate (R = OMe, OEt, OPr$^i$) /28-32/. The crystal structures show the lanthanide ion bound to eight sulphur atoms with coordination geometries ranging between the regular $D_{2d}$ dodecahedron, favored by alkoxy substituents at the phosphorus, and a dodecahedron distorted towards the $D_2$ square antiprism, favored by alkyl substituents. However for each ligand no structural change was observed along the lanthanide series in the solid state.

$^{31}P$ and $^{1}H$ LIS data have been reported for these $[Ln(S_2PR_2)_4]^-$ complexes in $CD_2Cl_2$ solution (Ln = Ce-Yb, except Pm and Gd), at 299 K for all complexes /18,28,32/ and also at 233 K for R = OEt ($S_2P(OEt)_2^-$ = O,O′-diethyl dithiophosphate) /28/. The observed NMR data fit an effectively axial symmetric coordination model in which the lanthanide ion is chelated by four $S_2PR_2^{2-}$ molecules in a bidentate fashion (through the two sulfur atoms). These data were analysed using the usual two-nuclei crystal field independent method /18/ through plots based on a simplified version of eq. (4), where $i$ is the $^{31}P$ nucleus and $k$ are the $CH_2$ and $CH_3$ protons and assuming no contact contribution to the LIS values of these protons. In this case, as $F_k = 0$, the plots give directly as intercepts the values of the hyperfine coupling constant to the $^{31}P$ nucleus, $F_P$, and as slopes the geometric ratios $R_{Pk} = G_P/G_k$. The breaks observed in such plots at the middle of the lanthanide series for all the compounds studied were assigned to structural ($R_{Pk}$ values) and $^{31}P$ coupling constant ($F_P$) changes along the lanthanide series, although no differences in solid state structures of the complexes along the series have been detected /18,28,32/. Large decreases of $F_P$ and $R_{Pk}$ values were observed from the first to the second group of Ln ions, indicating a substantial decrease of the contact and dipolar shifts of the $^{31}P$ nucleus, consistent with a change of the coordination polyhedron in solution from a dodecahedral to a square antiprismatic structure. We have confirmed the conclusions of these early studies by plotting the LIS data of the $[Ln(S_2P(OEt)_2)_4]^-$ complexes at 299 K and 233 K using eqs. (2) and (3) /26/. Indeed, breaks in those plots were seen at Tb, reflecting variations of the $F_i$ and $B_0^2 G_i$ terms (Table 1) /26/. The proton $F_i$ values are very small, while the $F_P$ and $R_{Pk}$ values indeed decrease drastically at Tb. In particular, the large $F_P$ couplings decrease by about 50% in the second part of the Ln series.

Because another early analysis of the same $^{31}P$ and $^{1}H$ LIS data using a different crystal field independent method has concluded that the $[Ln(S_2P(OEt)_2)_4]^-$ complexes are isostructural along the Ln series /14/, we proceeded to analyse the same data on the basis of the full eq. (4) /26/. The corresponding plots for $i = CH_3$ and $k = CH_2$ indeed give a single straight line identical at both temperatures, indicating that either there is no structural change, or if it occurs it is not reflected in the proton $G_i$ and $F_i$ parameters, eg. $G_{CH3}/G_{CH2}$ is constant (Fig. 2A). However, similar plots with $i$ = P and $k = CH_2$ or $CH_3$, identical to the ones discussed above using the simplified version of eq. (4), give clear breaks at Tb, confirming that the $F_i$ and $G_i$ parameters of the $^{31}P$ nucleus change abruptly at Tb (Fig. 2B). There is generally a good agreement of experimental and calculated $R_{ik}$ and $(F_i - F_k R_{ik})$ parameters (Table 1).

Plots according to eq. (5) with $i = CH_3$, $l = CH_2$ and $k$ = P give a single straight line along the Ln series (Fig. 2C), with experimental slope ($\alpha$) and intercept ($\beta$) values identical at both temperatures ($\alpha = 0.366$; $\beta = 0$) (Table 1). This is not surprising because the temperature-dependent parameters, $B_0^2$, $<S_z>$ and $C_j$, are all absent from eq. (5). The experimental $\alpha$ and $\beta$ parameters are in satisfactory agreement with those calculated from $F_i$ and $G_i$ values obtained by the other methods (Table 1). However, plots according to eq. (5) with other combinations, such as for $i$ = P, $l = CH_3$ and $k = CH_2$, show large breaks, thus confirming the structural and coupling constants change /26/. The reason why the first plot is not sensitive to the structural change is that $\beta = 0$ implies that $F_{CH2}/F_{CH3} = G_{CH3}/G_{CH2}$ and these ratios do not change along the series. This example illustrates how both the two nuclei and three nuclei methods may accidentally not reflect structural and/or $F_i$ changes for some of the combinations of nuclei used in the plots. Thus, it is very important to analyse all the possible combinations.

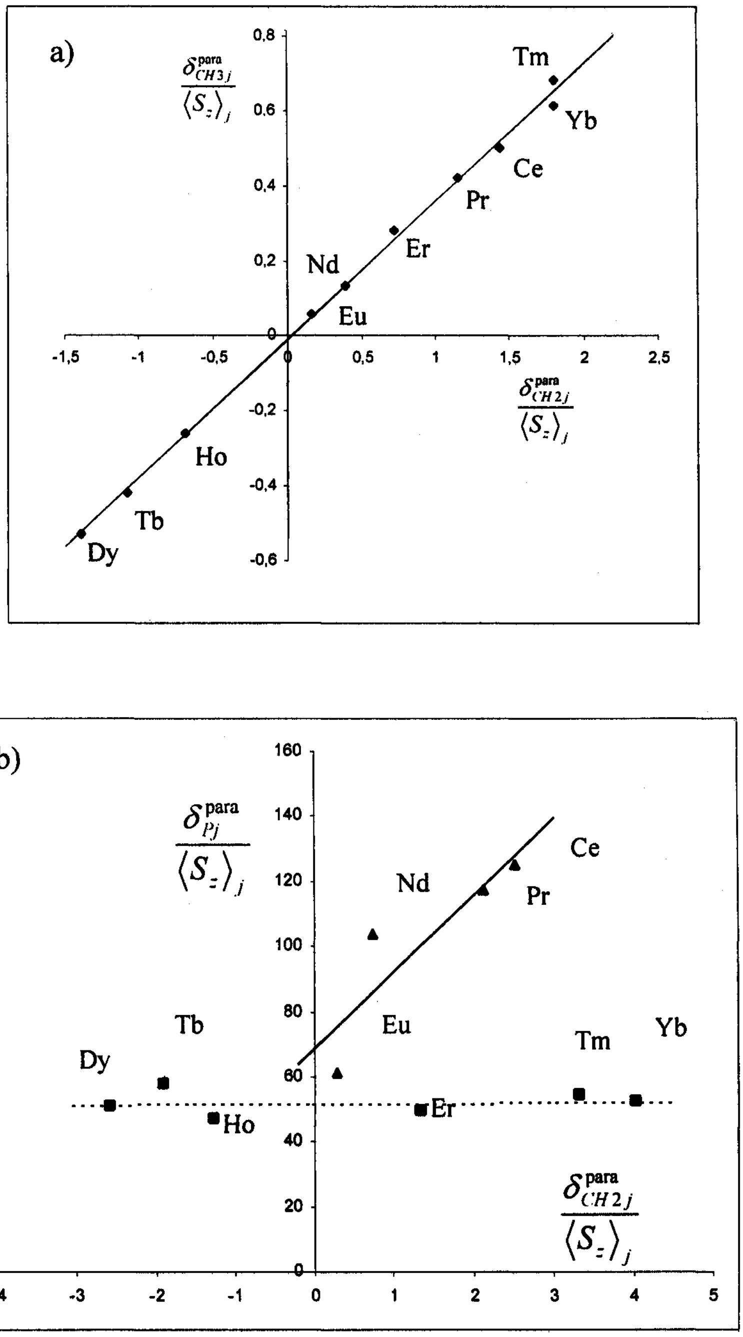

**Fig.2 :** Plots of $\frac{\delta_{ij}^{para}}{\langle S_z \rangle_j}$ vs $\frac{\delta_{kj}^{para}}{\langle S_z \rangle_j}$ a) for the $CH_3$-$CH_2$ pair; b) for the P- $CH_3$ pair; c) Plot of $\frac{\delta_{ij}^{para}}{\delta_{kj}^{para}}$ vs $\frac{\delta_{ij}^{para}}{\delta_{kj}^{para}}$ for the $CH_3$, $CH_2$, P triad at 253 K (Δ) and 296 K (♦). Data for $[Ln(P(^{OEt})_2S_2)_4]^-$ in $CD_2Cl_2$, 296 K (adapted from /26,28/).

**Fig. 2** continued

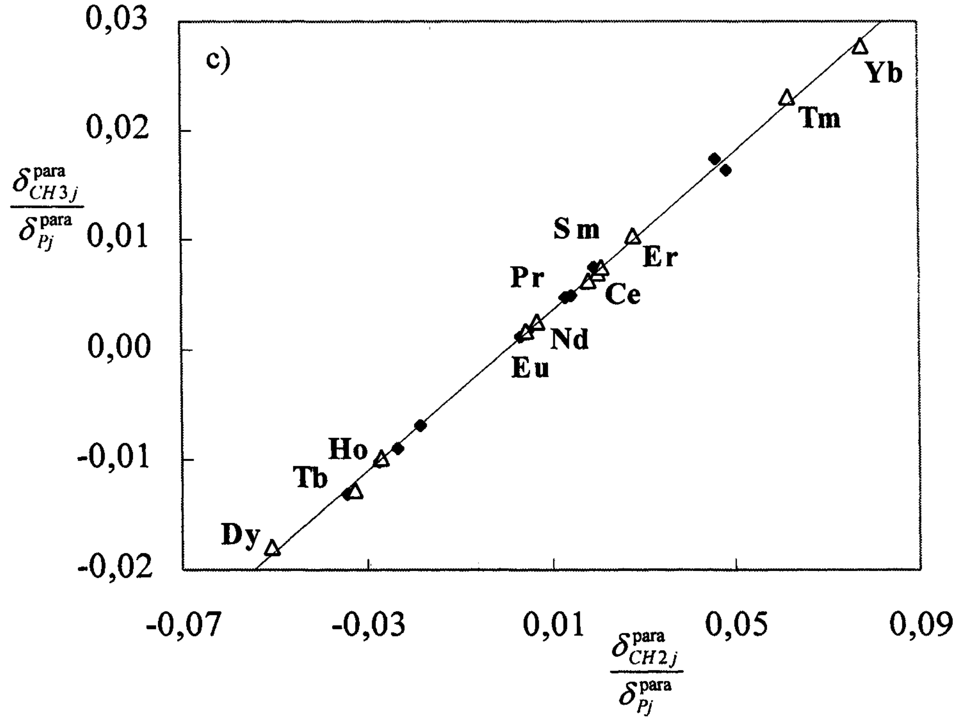

**$[Ln(DOTA)(H_2O)_q]^-$ ($L^2$ = DOTA = 1,4,7,10-tetraazacyclododecane-*N,N',N'',N'''*-tetraacetate)**

X-ray crystal structures have been determined for a series of $[Ln(DOTA)(H_2O)]^-$ chelates, which define nine–coordinated capped square antiprismatic (CSAP) (M) or inverted capped square antiprismatic structures (m). The acetate arms of the DOTA ligand are arranged in a propeller-like fashion above the basal plane containing one of the square faces of the coordination polyhedron, made up of the four ring N donor atoms which encompass the $Ln^{3+}$ ion, defining with their four O donor atoms the other square face in a parallel plane above it, thereby generating a $C_4$ symmetry axis in these complexes. The O atom of the inner-sphere water molecule occupies a capping position. The twist angle between these two square faces can be positive or negative, leading to the two possible isomers, M and m, in which the macrocyclic rings have the same conformation and the difference between them is in the layout of the pendant arms. The X-ray structures of the $[Ln(DOTA)(H_2O)]^-$ complexes with Ln = Eu, Gd, Y and Lu are CSAP (M), with twist angles of *ca* 39° /33-38/, whereas the La complex adopts an inverted CSAP structure (m) with twist angle of *ca* –22° /39/.

The solution structures of these complexes have been intensively studied by NMR. In fact, the $^1H$ and $^{13}C$ LIS values of the $[Ln(DOTA)]^-$ (Ln = La-Lu, except Pm and Gd) complexes have been reported in $D_2O$ at pH 7 and different temperatures /40-46/. The $^1H$ and $^{13}C$ NMR spectra exhibit two sets of resonances corresponding to the presence of two slowly interconverting coordination isomers, one set of resonances having constantly larger frequency shifts than the other group. The isomer displaying larger shifts was assigned to a nine-coordinate CSAP structure M, while the isomer displaying smaller shifts is either a nine-coordinate inverted CSAP m (from La to Ho), or an eight-coordinate inverted SAP m' structure with no

**Table 1**

Calculated values for $F_i$, $B_0^2 G_i$ and $R^2$ (linear correlation coefficient) values, according to eq. (2), comparison of $R_{ik}$ and $(F_i\text{-}R_{ik}F_k)$ parameters calculated directly according to eq. (4), and α and β parameters calculated directly according to eq. (5), with those obtained from the above $F_i$ and $B_0^2 G_i$ terms, for $^1$H- and $^{31}$P-nuclei in the $[Ln(S_2P(OEt)_2)_4]^-$ complexes in $CD_2Cl_2$ (adapted from /26,28/) ($^a$ Not determined.).

| | | T= 253 K | | | T = 296 K | | |
|---|---|---|---|---|---|---|---|
| Compd | | $CH_2$ | $CH_3$ | P | $CH_2$ | $CH_3$ | P |
| R= Ce-Eu | $F_i$ | 0.35 | 0.12 | 56.43 | 0.18 | 0.07 | 49.38 |
| | $B_0^2G_i$ | 0.37 | 0.13 | 22.65 | 0.21 | 0.07 | 14.83 |
| | $R^2$ | 0.928 | 0.920 | 0.923 | 0.950 | 0.931 | 0.962 |
| Compd | | $CH_2$ | $CH_3$ | P | $CH_2$ | $CH_3$ | P |
| R= Tb-Yb | $F_i$ | -0.35 | -0.13 | 49.90 | -0.23 | -0.09 | 38.90 |
| | $B_0^2G_i$ | 0.56 | 0.20 | -0.10 | 0.28 | 0.10 | -0.48 |
| | $R^2$ | 0.988 | 0.986 | 0.994 | 0.967 | 0.947 | 0.994 |

| Compd | | $CH_3-CH_2$ | P-$CH_3$ | $CH_3-CH_2$ | P-$CH_3$ |
|---|---|---|---|---|---|
| R= Ce-Eu | $R_{ik}$(exp) | 0.36 | a | 0.37 | 86.03 |
| | $(F_i\text{-}R_{ik})$(exp) | -0.01 | a | -0.01 | 56.31 |
| | $R^2$ | 0.999 | a | 0.998 | 0.826 |
| | $R_{ik}$(calc) | 0.34 | a | 0.35 | 200.4 |
| | $(F_i\text{-}R_{ik})$(calc) | 0.00 | a | 0.00 | 36.16 |
| R= Tb-Yb | $R_{ik}$(exp) | 0.37 | a | 0.37 | -3.31 |
| | $(F_i\text{-}R_{ik})$(exp) | -0.01 | a | -0.01 | 39.81 |
| | $R^2$ | 0.998 | a | 0.998 | 0.276 |
| | $R_{ik}$(calc) | 0.35 | a | 0.37 | -1.07 |
| | $(F_i\text{-}R_{ik})$(calc) | 0.00 | a | -0.01 | 38.74 |

| Compd | | $i = CH_3$; $k$ = P ; $l = CH_2$ | $i = CH_3$; $k$ = P ; $l = CH_2$ |
|---|---|---|---|
| R= Ce-Eu | α (exp) | 0.36 | 0.36 |
| | β (exp) | 0.00 | 0.00 |
| | $R^2$ | 0.999 | 0.999 |
| | α (calc) | 0.34 | 0.35 |
| | β (calc) | 0.09 | 0.00 |
| R= Tb-Yb | α (exp) | 0.36 | 0.36 |
| | β (exp) | 0.00 | 0.00 |
| | $R^2$ | 0.999 | 0.999 |
| | α (calc) | 0.37 | 0.37 |
| | β (calc) | 0.00 | 0.00 |

inner-sphere water molecule (q = 0) (Er to Lu), with varying relative populations along the Ln series /41,42,45/. It should be noted that the structures determined in the solid state contain two structurally independent elements of chirality defined by the pendant arm $C_4$-$C_3$-N-$C_1$ and ring N-$C_1$-$C_2$-N torsion angles, leading to four possible stereoisomers, which constitute two diastereoisomers each with enantiomeric pairs which are not distinguishable by NMR spectroscopy in solution. The numbering scheme adopted for the hydrogen and carbon atoms is shown in Fig. 3, which schematically represents part of the structure of the complexes in one of the enantiomeric forms of the M isomer, where $H_5$ denotes the pro-R and $H_6$ the pro-S pendant arm methylene proton /20/.

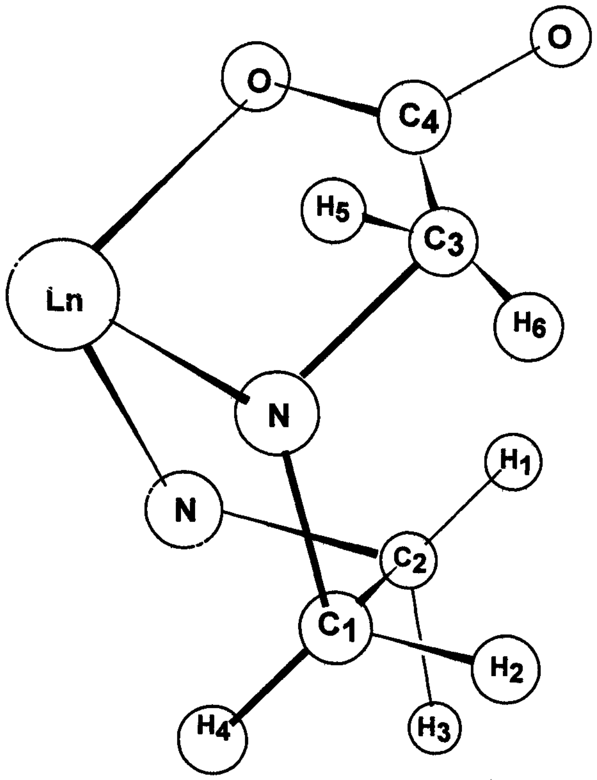

**Fig. 3:** Schematic model of a fragment of the structure of the M/M' isomer of a tetraazamacrocyclic Ln complex. Symmetry-related atoms are not shown for clarity. The numbering scheme for hydrogen and carbon/phosphorous atoms is also shown. $H_5$ denotes the pro-R and $H_6$ the pro-S pendant arm methylene proton (adapted from /20/).

The $^1H$ and $^{13}C$ LIS data available for the M, m and m' isomers of the $[Ln(DOTA)(H_2O_q]^-$complexes were analyzed by plots according to eqs. (2) and (3) /20/. Because Sm was excluded and due to population limitations, $^1H$ and $^{13}C$ LIS values of the M isomer were available in the first half of the Ln series only for Ln = Nd - Eu, and $^{13}C$ LIS values for the twisted SAP isomer were limited in the second half of the series to Yb (m'), limiting any definite conclusion in these cases. However, with the available data, breaks between light and heavy Ln were observed in most of those plots (Fig. 4), as reported previously /44/, reflecting variations of the $F_i$ and $B_0^2G_i$ parameters. In general, the M isomer shows less significant breaks than m/m'.

Systematic deviations were also observed for Tm and Yb from the linear correlations defined by the other $Ln^{3+}$ ions within the second half of the series.

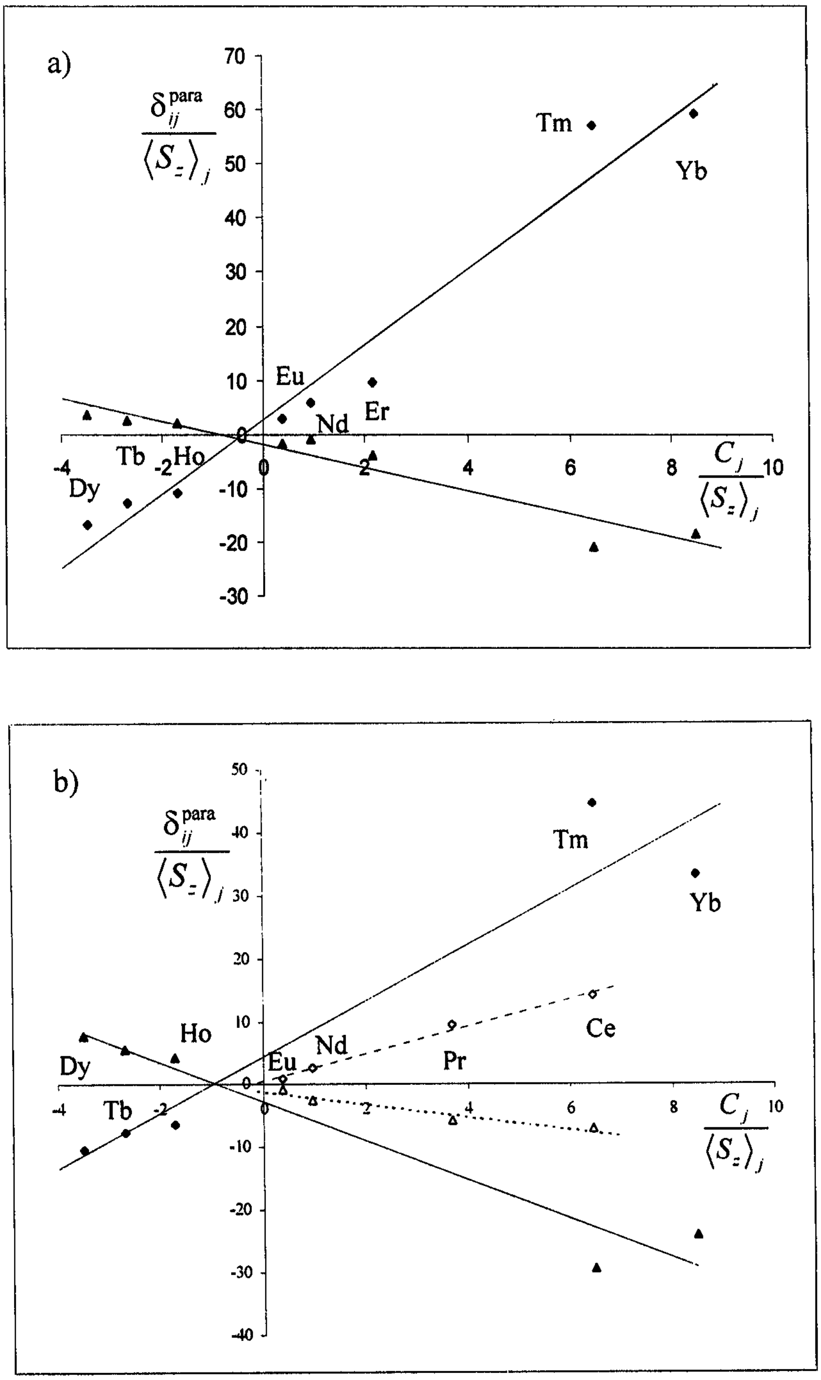

**Fig. 4:** Plots of $\frac{\delta_{ij}^{para}}{\langle S_z \rangle_j}$ vs $\frac{C_j}{\langle S_z \rangle_j}$ for $H_4$ (♦) and $H_6$ (▲) of a) M isomer (R= Nd-Yb); b) m/m' isomer (R= Ce-Yb) of $[Ln(DOTA)]^-$, $D_2O$, pH 7 (adapted from /26,44/).

The LIS data were also plotted according to eq. (4), eliminating the effect of any changes of $B_0^2$ (Fig. 5A) /20/. These plots again often show breaks at Eu/Tb, reflecting changes of $F_i$ and $R_{ik}$, and the $R_{ik}$ and $(F_i\text{-}R_{ik}F_k)$ parameters of the two groups (Ce-Eu and Tb-Yb) were evaluated. These breaks are much less significant than for the one-nucleus plots, not only due to the absence of $B_0^2$ in the later plots, but also due to the presence of geometric term ratios $R_{ik}$, which may be significantly less affected by small structural effects on $G_i$ values

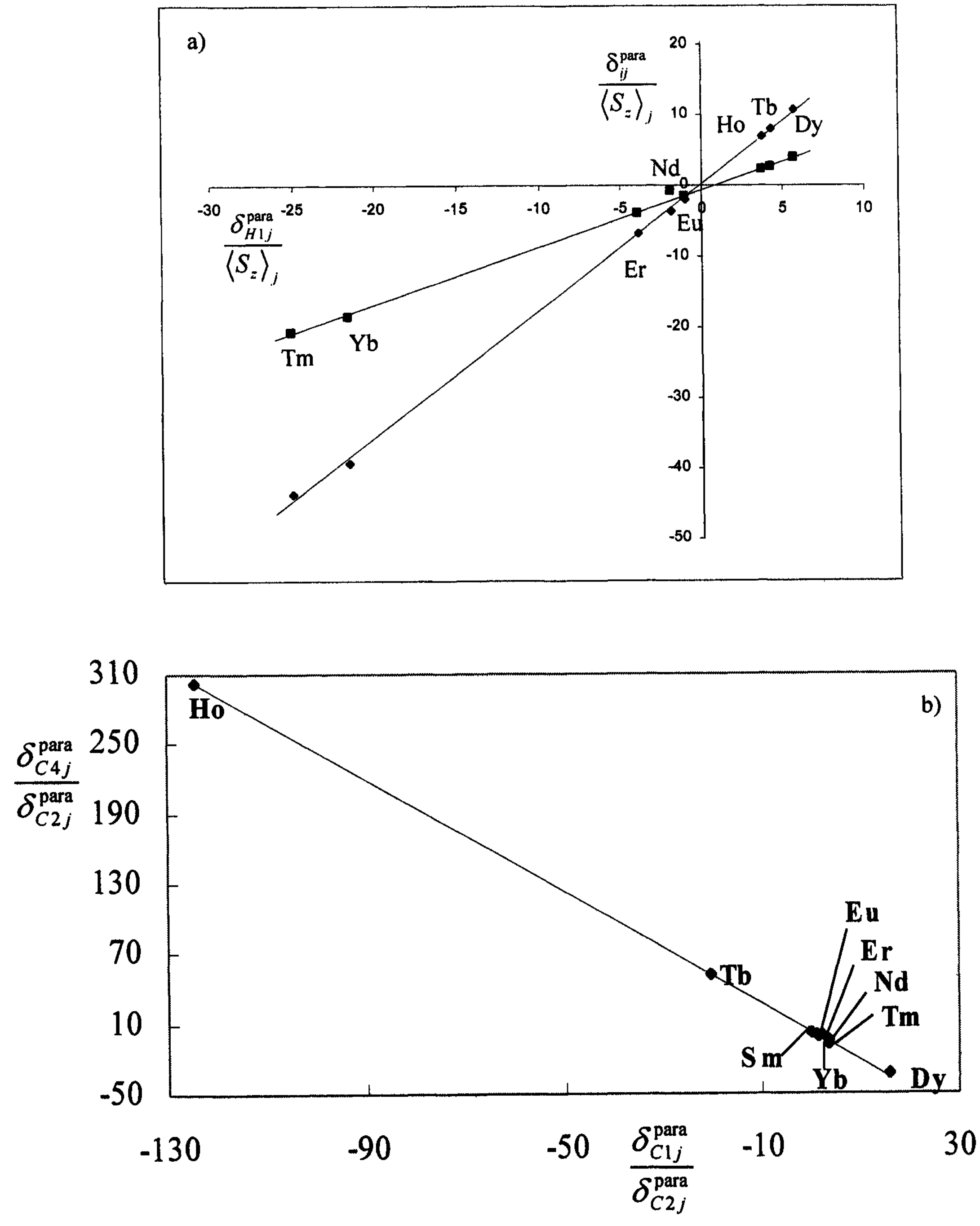

Fig. 5 continued

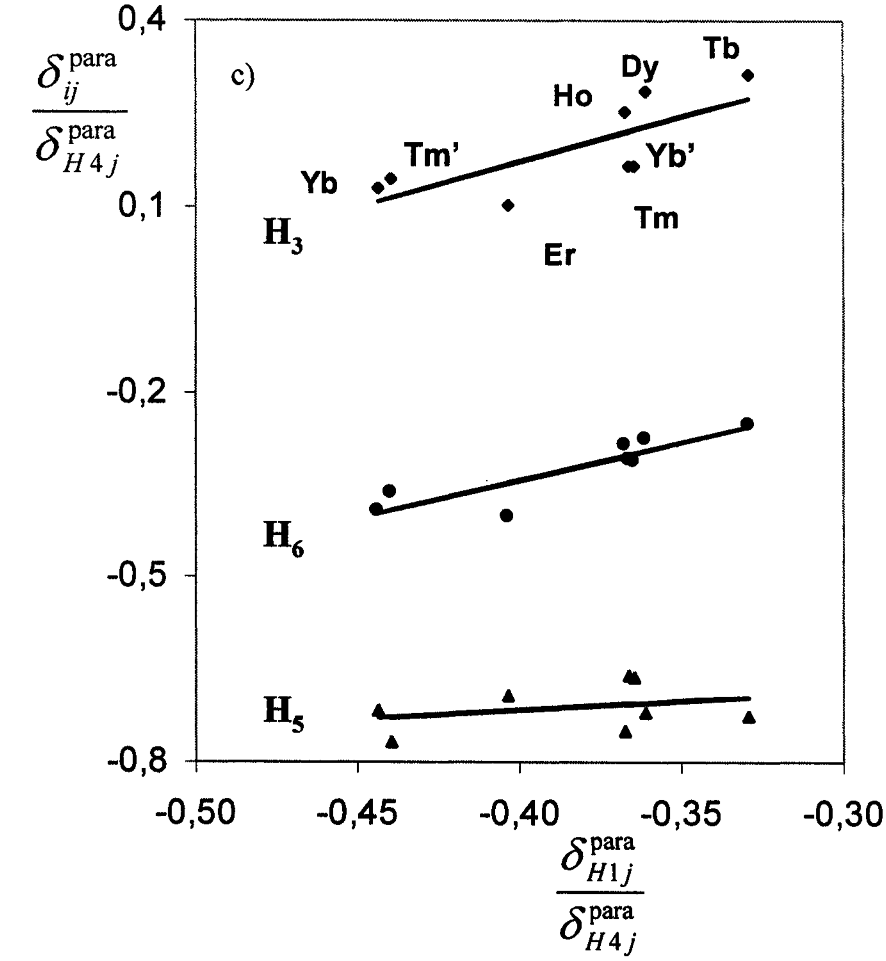

**Fig. 5:** a) Crystal-field independent plots of $\frac{\delta^{para}_{ij}}{\langle S_z \rangle_j}$ vs $\frac{\delta^{para}_{kj}}{\langle S_z \rangle_j}$ for the $H_5$-$H_1$ (♦) and $H_6$-$H_1$ (■) pairs of the M isomer; b) Plots of $\frac{\delta^{para}_{ij}}{\delta^{para}_{kj}}$ vs $\frac{\delta^{para}_{lj}}{\delta^{para}_{kj}}$ for the $C_4$, $C_1$, $C_2$ triad of the M isomer; c) for the $H_i$, $H_1$, $H_4$ triads ($H_i = H_3$ (♦), $H_5$ (▲), $H_6$ (•)) of the m/m' isomer, except Tm' and Yb', which are from the M isomer; $[Ln(DOTA)]^-$, $D_2O$, pH 7 (adapted from /20, 26/).

due to the effect of lanthanide contraction /20/. However, the breaks observed are statistically significant for $H_4$, $H_3$, $H_2$, $C_4$ and $C_2$. The $R_{ik}$ and $(F_i$-$R_{ik}F_k)$ parameters evaluated directly by eq. (4) agree very well with those obtained indirectly using the $F_i$ and $B_0^2G_i$ values from eq. (2) /20/.

Various plots using eq. (5) were also obtained for this system /26/. For the $^{13}C$ shifts of the M isomer, some of the plots give good linear correlations along the Ln series, eg. for $i = C_3$ or $C_4$, $l = C_1$ and $k = C_2$ (Fig. 5B). Other plots of this type give again a single line along the Ln series, such as for $i = C_2$, $l = C_1$ and $k = C_3$, but other combinations give more or less pronounced breaks, such as for $i = C_4$, $l = C_1$ and $k = C_3$, and for $i =$

$C_2$ or $C_3$, $l = C_1$ and $k = C_4$ /26/. This is in agreement with the changes of $F_i$ and $G_i$ parameters at the middle of the Ln series detected by the one- and two nuclei methods, and again illustrates the possibility that some of the plots according to eq. (5) may accidentally be linear without implying isostructurality of the complexes. In the case of the $^{13}C$ shifts of the m isomer, the data available (Ln = Pr, Nd, Eu) gives linear plots within the first half of the Ln series (see Table 2). Plots according to eq. (5) for the $^1H$ LIS data of the M and m isomers, eg. for $i = H_3$, $H_5$ and $H_6$, $l = H_1$ and $k = H_4$ support these conclusions, showing two linear parts with breaks in the middle of the series (Fig. 5C). Table 2 compares the α and β values calculated for the two isomers, clearly showing that M has significantly larger values than m/m' /26/. The structural change occurring at Ho for the inverted SAP isomer, from m to m', involving loss of one hydration water molecule, is not reflected in any break at Ho in the plots obtained.

**Table 2**

Comparison of the α and β parameters for the $^1H$ LIS of the Ln-tetraazamacrocyclic complexes $[Ln(DOTA)]^-$ (M and m), $[Ln(DOTEA)]^{3+}$ and $[Ln(DOTP)]^{5-}$ obtained using the graphical method based on eq. (5) (adapted from /26/).

| Ln(III) complex | $i = H_2$ | | $i = H_3$ | | $i = H_5$ | | $i = H_6$ | |
|---|---|---|---|---|---|---|---|---|
| $k = H_4$; $l = H_1$ | α | β | α | β | α | β | α | β |
| **Ln = Ce – Eu** | | | | | | | | |
| $[Ln(DOTP)]^{5-}$ | 1.07 | 0.56 | 0.99 | 0.47 | 1.25 | -0.278 | 0.64 | -0.05 |
| $[Ln(DOTA)]^-$ (M) | a | a | a | a | a | a | a | a |
| $[Ln(DOTA)]^-$ (m) | 1.48 | 0.44 | 1.88 | 0.74 | 2.20 | 0.22 | 0.40 | -0.12 |
| $[Ln(DOTEA)]^+$ | 14.09 | 4.94 | 17.40 | 6.01 | -0.35 | -0.72 | 8.01 | 2.42 |
| **Ln = Tb –Yb** | | | | | | | | |
| $[Ln(DOTP)]^{5-}$ | 1.07 | 0.56 | 0.99 | 0.47 | 1.25 | -0.28 | 0.64 | -0.05 |
| $[Ln(DOTA)]^-$ (M) | 3.11 | 1.28 | 2.17 | 0.97 | 0.80 | -0.37 | 3.00 | 0.80 |
| $[Ln(DOTA)]^-$ (m) | 0.86 | 0.60 | 1.54 | 0.80 | -0.01 | -0.72 | 1.25 | 0.16 |
| $[Ln(DOTEA)]^+$ | 1.40 | 0.78 | 1.91 | 0.98 | 0.16 | -0.64 | 1.41 | 0.27 |

[a] Not determined.

### $[Ln(DOTEA)]^{3+}$ ($L^3$ = DOTEA = 1,4,7,10-tetraazacyclododecane-1,4,7,10-tetrakis(*N,N*-diethylacetamide))

Although there is no crystal structure available for any of the $[Ln(DOTEA)]^{3+}$ complexes (DOTEA is the DOTA-like tertiary tetraethylamide derivative), there are crystal structures available for $Ln^{3+}$ complexes for various DOTA-like achiral primary and secondary tetramide derivatives. Some of these structures are m-type for $[La(DOTAM)(H_2O)]^{3+}$ /47/ and $[Eu(DOTAM)(H_2O)]^{3+}$ /48/, and M-type for $[Ln(DTMA)(H_2O)]^{3+}$ (Ln = Gd, Dy) /49,50/. However, the m/M isomer ratio for $Eu^{3+}$ complexes of various DOTA tetraamide derivatives in solution increases in the order tetraacetate ($[Eu(DOTA)]^-$) < primary tetraamide ($[Eu(DOTAM)]^{3+}$) < secondary tetramethylamide ($[Eu(DTMA)]^{3+}$) < tertiary tetramethylamide ($[Eu(DOTTA)]^{3+}$), indicating that an increasing steric demand at the bound metal ion favors the inverted square antiprismatic structure m /50/.

$^1$H and $^{13}$C LIS values are available for the $[Ln(DOTEA)]^{3+}$ complexes (Ln = Ce-Yb except Pm and Gd, for $^1$H, and Ln = Pr, Nd, Sm, Eu for $^{13}$C) in $CD_3CN$ solution at 253K, which is present as a single isomer /51/. These $^1$H LIS data were originally analysed using the one-nucleus method, through plots according to eqs. (2) and (3), which showed breaks at Eu/Tb, reflecting variations of the $F_i$ and $B_0^2G_i$ parameters, while the data for Tm significantly deviated from the linear correlation defined by the (Tb-Yb) group. The calculated $B_0^2G_i$ differed by 30% for the two Ln subgroups, while the $F_i$ differed by 600% /51/. The $^1$H LIS data were also analysed by comparing the experimental dipolar shifts, which are equal to the z-axis magnetic anisotropy $C_j B_0^2 G_i$, with those calculated from the $G_i$ values defined by MM2-calculated structures of these complexes. The optimal calculated structure was of the M type, and the results further suggested a significant difference in $G_i$ values between the light and heavy complexes. However, the derived $C_j B_0^2 G_i$ values for the series of $[Ln(DOTEA)]^{3+}$ complexes did not follow the trend of $C_j$ constants, which was interpreted as due to a change of the $B_0^2$ parameter along the Ln series, with the largest value for Tm /51/. However this analysis, which led to an M-type solution structure of these complexes, is in conflict with the solution structure analysis of the other tertiary tetraamide complex $[Eu(DOTTA)]^{3+}$, with a m/M isomer ratio of 2 /50/.

We reanalyzed these $^1$H LIS data, as well as the $^{13}$C LIS data (R= Pr, Nd, Eu, excluding Sm) for the $[Ln(DOTEA)]^{3+}$ complexes, through plots according to eqs. (2) and (3) /20/ and using the $H_5$ and $H_6$ assignments of Fig. 3, leading to some reassignments of the original data /51/. Again breaks were observed at Eu/Tb in most of those plots, reflecting variations of $F_i$ and $B_0^2G_i$ /20/. The data were also plotted according to eq. (4) (Fig.6A), showing again much smaller ($H_5$, $H_6$ << $H_4$ < $H_2$, $H_3$.), but still significant breaks at

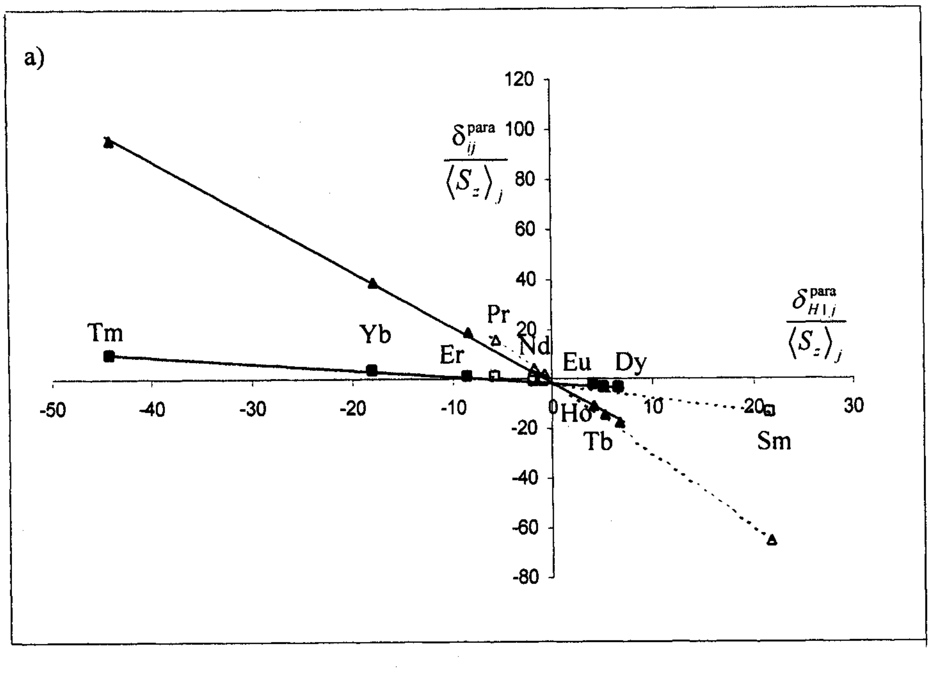

Fig. 6 continued

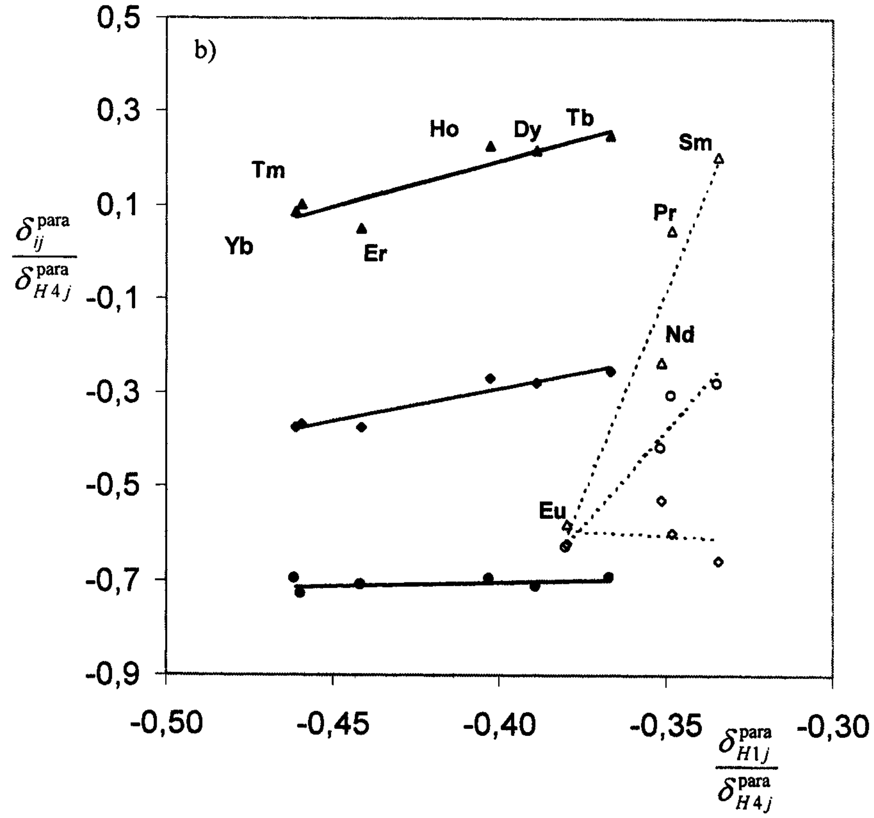

**Fig. 6:** a) Plots of $\frac{\delta_{ij}^{para}}{\langle S_z \rangle_j}$ vs $\frac{\delta_{kj}^{para}}{\langle S_z \rangle_j}$ for the $H_2$-$H_1$ (♦) and $H_3$-$H_1$ (■) pairs; b) Plots of $\frac{\delta_{ij}^{para}}{\delta_{kj}^{para}}$ vs $\frac{\delta_{lj}^{para}}{\delta_{kj}^{para}}$ for the $H_i$, $H_l$, $H_4$ triads ($H_i = H_3$ (▲), $H_5$ (•), $H_6$ (♦)) of $[Ln(DOTEA))]^{3+}$, $D_2O$, pH 7 (adapted from /20, 26/).

Eu/Tb, confirming the changes of $F_i$ and $R_{ik}$ /20/.

Fig. 6B shows $^1$H LIS data plotted according to eq. (5) for $i = H_3$, $H_5$ and $H_6$, $l = H_1$ and $k = H_4$, which again exhibit a break at Eu/Tb, thereby confirming the $F_i$ and $G_i$ changes (see Table 2 for the calculated α and β values) /26/.

### $[Ln(DOTP)]^{5-}$ ($L^4 = DOTP^{8-}$ = 1,4,7,10-tetraazacyclododecane-1,4,7,10-tetrakis (methylenephosphonate))

The crystal structure of the $[Tm(DOTP)]^{5-}$ complex describes the $Tm^{3+}$ coordination polyhedron as an inverted square antiprism, m', with the four ring nitrogens defining one of its square faces and the four coordinated phosphonate oxygens defining the other one /52/. Structurally very similar to the $[Ln(DOTA)]^-$ complexes, except for the absence of inner-sphere water molecules, it also has a $C_4$ symmetry axis. The $^1$H, $^{13}$C and $^{31}$P LIS for the paramagnetic $[Ln(DOTP)]^{5-}$ complexes (except Ln = Pm, Gd) (where $DOTP^{8-}$ is the tetakis(methylenephosphonate) analog of DOTA) have been reported at 298 K in $D_2O$ at pH 10 /53/, showing only one isomer in solution (M' or m'). Water $^{17}O$ NMR shift measurements of $[Dy(DOTP)]^{5-}$ confirmed that this complex lacks an inner-sphere water molecule /16/. The highly charged anionic $[Ln(DOTP)]^{5-}$ complexes have four protonation steps over the pH 3-10 range, leading to significantly pH dependent LIS values /54/. Thus, besides pH 10, the LIS values at pH 7 and 3 were also analyzed /20/. These four protonations correspond to the four residual negative charges localized on the phosphonate oxygen atoms, which are directed away from the Ln coordination site, while the fifth charge, which is averaged over the four bound oxygens in the coordination cage, is not titrated in this pH range.

The $^{1}H$, $^{13}C$ and $^{31}P$ LIS data (pH 10) were initially analysed through the one-nucleus technique. Shift data plots according to eqs. (2) and (3) /53/ showed breaks at Eu/Tb, reflecting variations of $F_i$ and $B_0^2G_i$. Systematic deviations were again observed for Tm and Yb from the linear correlations defined by the other ions within the second half of the series. The $^{1}H$ LIS data were also analysed by considering $B_0^2$ constant and comparing the experimental $G_i$ values and Ln-$H_i$ distance ratios obtained from $T_1$ relaxation data, with the corresponding values derived from MMX-calculated structures of these complexes, giving an optimal fit for an M'-type structure, in disagreement with the crystal structure /53/.

Because these contradictory structural results might originate from the assignments of the methylene protons of the pendant arms, in a recent reinvestigation, the $H_5$ and $H_6$ protons were reassigned to be in agreement with the convention of Fig 3, and the $^{1}H$ and $^{13}C$ LIS data available for the $[Ln(DOTPH_n)]^{(4n-5)}$ complexes at pH 10, 7 and 3 were reanalyzed /20/. Plots according to eqs. (2) and (3) again showed breaks at Tb, due to $F_i$ and $B_0^2G_i$ changes /20/. Plots according to eq. (4) again show smaller ($H_5$, $H_6 << H_4$, $C_3 < H_2$, $H_3$, $C_2$, P) but significant breaks at Tb, indicating changes of $F_i$ and $R_{ik}$ at Eu/Tb /20/. An earlier analysis of the $^{1}H$, $^{13}C$ and $^{31}P$ LIS data for the $[Ln(DOTP)]^{5-}$ complexes within the second half of the series (Ln = Tb-Yb) /16/ using Reuben's crystal-field parameter independent method /14/ gave evidence that these complexes are isostructural ($G_i$ and $F_i$ constant). It also showed that the deviations in the plots according to eqs. (2) and (3) is often observed in the (Tb-Yb) half series, in particular for Tm and Yb, reflect large changes of $B_0^2$, with the largest value for Tm and the smallest for Yb /16/.

Fig. 7A shows the $^{31}P/^{13}C$ LIS data plotted according to eq. (5) for $i$ = P, $l = C_1$ and $k = C_2$, where all data points (n=11) fall on a straight line. The $^{13}C$ LIS data of $C_3$ when plotted in the same way (i= P, $l = C_1$ and $k = C_2$ ) also show a good linear relationship /26/. The α and β values obtained directly from these plots agree quite reasonably with those calculated from the $F_i$ and $B_0^2 G_i$ parameters obtained from eq (2) /26/. However, like for the complexes of DOTA and DOTEA, plots using other nuclear combinations give more or less pronounced breaks, such as for $i = C_4$, $l = C_1$ and $k = C_3$, and for $i = C_2$ or $C_3$, $l = C_1$ and $k = C_4$. This is again in agreement with the detected changes of $F_i$ and $G_i$ parameters at Eu/Tb, and illustrates the appearance of accidentally linear plots according to eq. (5). These conclusions are supported by plots of the $^{1}H$ LIS data according to eq. (5), obtained for $i = H_2$, $H_3$, $H_5$ and $H_6$, $l = H_1$ and $k = H_4$. While the plots are reasonably linear for $i = H_5$ and $H_6$ (Fig. 7B), they show large deviations from linearity for $i = H_2$ and $H_3$ (see α and β values at Table 2) /26/.

Due to the conflicting conclusions from the structural analysis in solution and in the solid state for some of the tetraazamacrocyclic complexes studied ($[Ln(DOTEA)]^{3+}$ and $[Ln(DOTP)]^{5-}$), described above, we compared the experimental proton structural parameters, $R_{kl}$ (dipolar shift ratios) and ($r_{Hj}/r_{H1}$) (Ln-$H_i$ distance ratios) with those calculated from M/M′ and m/m' structural models in the four systems studied (M

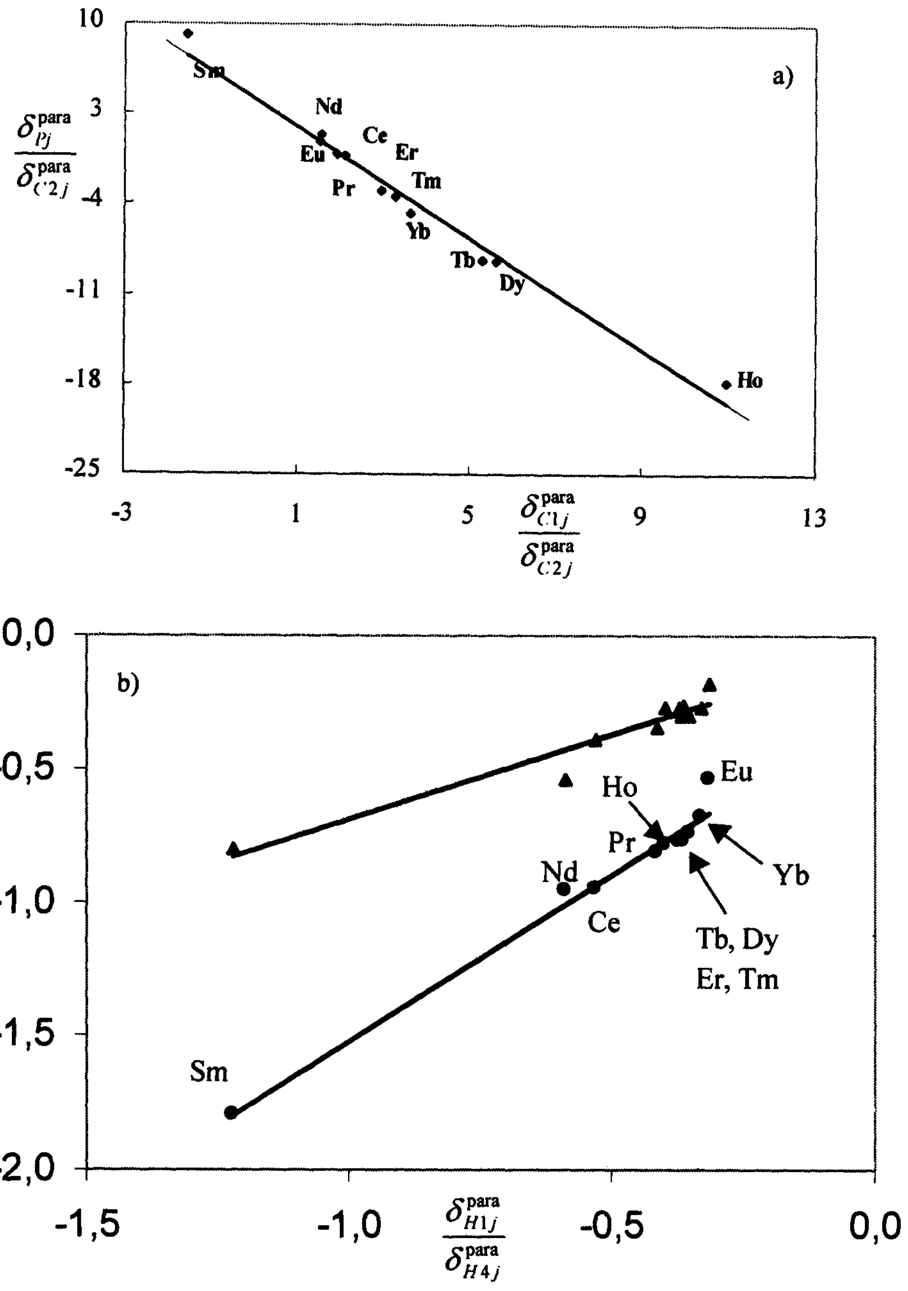

**Fig. 7:** Plots of $\frac{\delta^{para}_{ij}}{\delta^{para}_{kj}}$ vs $\frac{\delta^{para}_{lj}}{\delta^{para}_{kj}}$ a) for the P, $C_1$, $C_2$ triad; b) for the $H_i$, $H_1$, $H_4$ triads ($H_i$ = $H_5$ (•), $H_6$ (♦)) of $[Ln(DOTP)]^{5-}$, $D_2O$, pH 10 7 (adapted from /26/).

and m isomers of $[Ln(DOTA)]^-$, $[Ln(DOTEA)]^{3+}$ and $[Ln(DOTP)]^{5-}$) /20/. The $R_{kl}$ ratios are quite constant for the ring protons in all complexes, while the differences between the M/M′ and m/m' structures occur in the $H_5$ and $H_6$ protons of the pendant arms. Very good agreement of experimental and calculated data was obtained for the M and m $[Ln(DOTA)]^-$ isomers (also with the X-ray results) and for the $[Ln(DOTEA)]^{3+}$ complexes in the M form. For the $[Ln(DOTP)]^{5-}$ complexes, the reassignment of $H_5$ and $H_6$ gave optimal agreement for the m' solution conformation, in accordance with the X-ray crystal structure /20/. A similar comparison, using the experimental Yb-H distances normalized to $H_1$ ($r_{Hj}/r_{H1}$) obtained from the proton $T_1$ relaxation times in the literature /41,51,53/ and eq (6), with those calculated from M/M′ and m/m' structural models, confirms the above conclusions. The Yb-$H_i$ distances of $H_5$ and $H_6$ in the M/M' and m/m' forms differ quite substantially: while $H_5$ is closer to Yb than $H_6$ in M, their distances to Yb are about the same in m/m'. The experimental results for the $[Ln(DOTA)]^-$ M and m/m' isomers agree very well with the predicted values, the experimental results for the $[Ln(DOTEA)]^{3+}$ complex agree with an M form in solution, and the experimental data for $[Ln(DOTP)]^{5-}$ also agree with the values calculated for a m' form /20/.

It is worth noting that comparison of the α and β values, obtained from plots of the $^1H$ LIS for the $CH_2$ protons in the pendant arms of the three complexes according to eq. (5), for $i = H_5$ and $H_6$, with $l = H_1$ and $k = H_4$ (Table 2), is not diagnostic of their structure being either M/M' or m/m', although these structures only differ in the arrangement of the pendant arms around the metal ion. The α and β parameters do not reflect those structural changes, probably as a result of their very indirect structural dependence on the $R_{iH4}$ ($i = H_5$ and $H_6$) geometric ratio (eq. (5)), mixed with a dependence on the geometric ratio, $R_{H1H4}$, and the $F_i$ ratios, $S_{iH4}$ and $S_{H1H4}$, which significantly change in the different complexes. This reduces the structural diagnostic power of the three nuclei method based on eq. (5) /20, 26/.

### $[Ln(PHT)_2]^-$ ($L^5$ = PHT = phtalocyanine).

Several X-ray crystallographic studies on $Nd^{3+}$ /55/ and $Lu^{3+}$ /56/ sandwich complexes of $Ln^{3+}$ ions with two phtalocyanine (PHT) macrocyclic conjugated rings have been carried out (H $[Nd(PHT)_2]^-$ $[N(n\text{-}Bu)_4]]$ $[Lu(PHT)_2]$ and H $[Lu(PHT)_2]$) These solid-state structures are very similar, with the $Ln^{3+}$ eight-coordinated by the isoindole nitrogen atoms of the two PHT rings in quasi square antiprismatic geometries ($D_{4d}$ symmetry), with the two stacked phtalocyanines staggered.

The $^1H$ NMR spectra of the $[N(n\text{-}Bu)_4]]$ $[Ln(PHT)_2]$ (Ln = Pr-Lu except Pm, Gd) complexes in $CD_3CN$ solution at 298 K show only one $H_1$ and one $H_2$ resonance of the phtalocyanine rings, indicating that the complexes have a $C_4$ axis in solution /57/. This is compatible with a $D_{4h}$ square prismatic or a $D_{4d}$ square antiprismatic geometry, depending whether the two stacked phtalocyanines are eclipsed or staggered, or fast interconversion between the two. Data from the UV-vis absorption spectra of the Ln = Pr, Lu complexes in $CD_3CN$ solution excluded the $D_{4h}$ structure /57/. The LIS values of the $H_1$ and $H_2$ protons in the paramagnetic complexes were analysed through plots according to eqs. (2) and (3), showing breaks at Eu/Tb and also significant deviations from linearity within each of the two Ln subfamilies, reflecting variations of $F_i$ and $B_0^2 G_i$ (Fig. 8A) /26/. In the original work, $B_0^2 G_i$ values were identified with $G_i$ and single $F_i$ and $G_i$ values were obtained for $H_1$ and $H_2$ along the lanthanide series /57/ which did not fully agree with our analysis /26/. Fitting of the geometrical dependence of the experimentally derived *Rik* ratios to a chemical model of the $[Ln(PHT)_2]^-$ complexes based on the crystal structures of the Nd and Lu compounds gave an average distance between the two rings in the sandwich compound of 2.54 Å and an average Ln-N distance of 2.31 Å /57/. However, the data analysis available does not prove isostructurality in solution.

Thus, we studied the same data using the two-nuclei crystal-field independent technique, eliminating the effect of any $B_0^2$ changes, and a plot according to eq. (4) ($i = H_2$ and $k = H_1$) gives a single straight line along the lanthanide series (Fig. 8B) /26/. This confirms that the complexes are isostructural, with change of $B_0^2$

and $F_i$ along the series. There is generally a good agreement of experimental and calculated $R_{ik}$ and $(F_i - F_k R_{ik})$ parameters /26/.

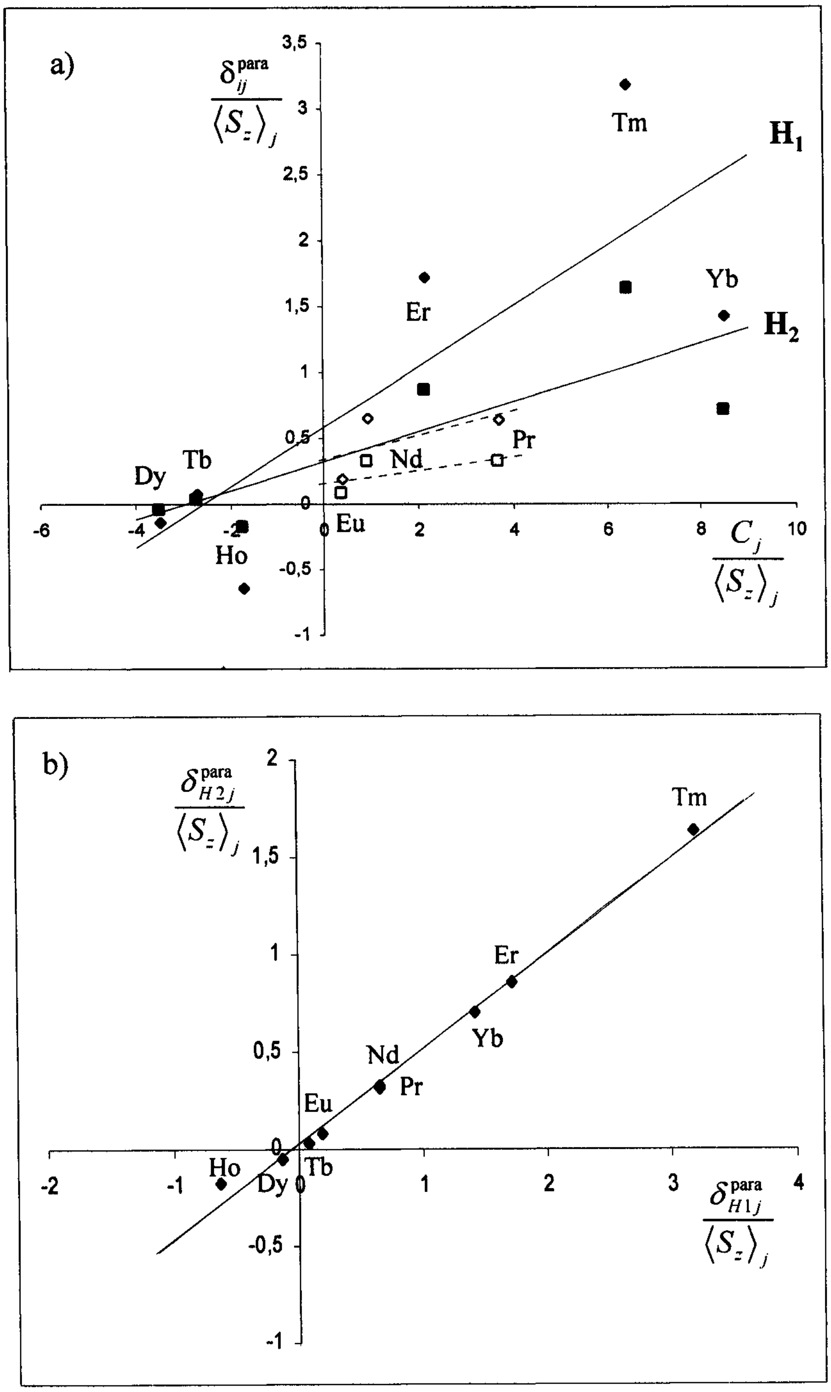

**Fig. 8:** a) Plots of $\frac{\delta^{para}_{ij}}{\langle S_z\rangle_j}$ vs $\frac{C_j}{\langle S_z\rangle_j}$ for $H_1$ (♦) and $H_2$ (■); b) Plot of $\frac{\delta^{para}_{ij}}{\langle S_z\rangle_j}$ vs $\frac{\delta^{para}_{kj}}{\langle S_z\rangle_j}$ for the $H_1$-$H_2$ (♦) pair; $[Ln(PHT)_2]^-$ in $CD_3CN$ (adapted from /26, 57/).

### CONCLUSIONS

The considerable success of the classical one-nucleus crystal-field dependent method in the separation of experimental $\delta_{ij}^{\text{para}}$ values for axially symmetric lanthanide complexes into their contact and dipolar contributions is somewhat limited in its structural information due to the observation of data scatter or breaks in the corresponding plots according to eqs. (2) and (3)) often seen for many systems. The resulting large statistical errors in $F_i$ and $B_0^2 G_i$ values obtained by the method often preclude any reliable quantitative study of the structural and bonding properties of the respective lanthanide complexes. In particular, the $B_0^2 G_i$ term obtained cannot be used for detecting structural $G_i$ changes because it is affected by any variation of the crystal-field parameter $B_0^2$ along the lanthanide series /1c/. The two-nuclei technique (eq. (4)) /18,19/, independent of $B_0^2$, is a reliable method to study the isostructurality of a series of lanthanide complexes. The linearity of the corresponding experimental plots is a proof of isostructurality of the complexes, due to the constancy of their slopes $R_{ik}=(G_i/G_k)$ and intercepts $(F_i-F_kR_{ik})$, and therefore of the geometric terms $G_i$ and hyperfine coupling constants $F_i$. The combined use of the one- and two-nuclei techniques allows us to conclude if the changes of $B_0^2 G_i$ reflected by breaks of plots using the first method result from changes of $B_0^2$, $G_i$ or both /20/. The three nuclei shift ratio method (eq (5)) /22/ has some advantages in the analysis of the isostructurality of lanthanide complexes, as it is based exclusively on the experimental shift data, requiring no knowledge of $B_0^2$, $\langle S_z \rangle$ or $C_j$ values, and thus can be applied to the shift data measured at any temperature, as long as the data are available for at least three nuclei within a given ligand. However, the $\alpha$ and $\beta$ values of the plots obtained are complicated functions of $F_i$ and $G_i$ ratios rather than of their values, which may reduce or magnify in some cases the effects of $Ln^{3+}$ contraction on $F_i$ and $G_i$ parameters. It also cannot provide quantitative values for $F_i$ and $G_i$.

The combined use of the three methods gives new insights for the solution structural study of a series of lanthanide complexes on the basis of the measured LIS values, as shown by a recent critical analysis of the LIS data for several series of $Ln^{3+}$ complexes of $C_3$ symmetry in terms of structural changes, crystal-field effects and/or variation of hyperfine constants along the lanthanide series /26,27/. In the present study, this approach was extended to a series of linear and macrocyclic $Ln^{3+}$ complexes of $C_4$ symmetry, at different temperatures, solvents and pH values. None of the systems studied showed constancy of the three $F_i$, $G_i$ and $B_0^2$ parameters for the whole series of lanthanide complexes, but isostructurality was proven, with change of $F_i$ and $B_0^2$, for the complexes of $L^5$ ($[Ln(PHT)_2]^-$) using the one- and two-nuclei methods. Sudden variations of the $F_i$, $G_i$ and $B_0^2$ parameters at Eu/Tb were observed for the series of complexes of all the other ligands studied, $L^1$-$L^4$. Breaks in plots according to the two-nuclei method were generally smaller than for the one-nucleus method, as the geometric term ratios $R_{ik}=(G_i/G_k)$ tend to be less sensitive than the geometric terms $G_i$ to the structural changes that may occur. For some particular combinations of nuclei, the plots according to eqs. (4) and (5) are accidentally linear, without necessarily implying isostructurality of the complexes, as they involve parameters insensitive to the structural changes which may occur. This illustrates the need to analyse as many plots as possible, as conclusions based on a small number of plots may be wrong. The changes involving the three $F_i$, $G_i$ and $B_0^2$ parameters were interpreted as reflecting small distortions of dodecahedral towards SAP structures ($L^1$, $[Ln(S_2PR_2)_4]^-$ complexes) or small geometric changes within SAP and inverted SAP structures ($L^2$, $[Ln(DOTA)]^-$; $L^3$, $[Ln(DOTEA)]^{3+}$; $L^4$, $[Ln(DOTP)]^{5-}$). These observed changes of the parameters could also result from a magnification, by the present graphical analysis, of the breaks expected from the gradual structural changes along the series due to the lanthanide contraction /13/. The structural change occurring for the $[Ln(DOTA)]^-$ isomer with an inverted SAP structure, from m to m' at Ho, involving loss of one hydration water molecule, is not reflected in any break at Ho in the plots obtained. The various α and β parameters, obtained from plots according to the three-nuclei method for the tetraazamacrocyclic complexes, were found not to be diagnostic of their structure being either M/M' or m/m'. They do not reflect such structural differences, due to their very indirect dependence on the relevant geometric terms.

### ACKNOWLEDGMENT

We thank Dr. J. Peters for useful discussions. Support of this research by grants from Fundação da Ciência e Tecnologia, Portugal, the Robert A. Welch Foundation (AT-584) and the NIH Biotechnology Research Program (RR02584) is gratefully acknowledged.

### REFERENCES

1. (a) C.F.G.C. Geraldes, in *NMR in Supramolecular Chemistry*, Ed. M. Pons, Kluwer Academic, Amsterdam, p. 133 (1999). (b) I. Bertini and C. Luchinat, *Coord. Chem. Rev.*, **150**, 1 (1996). (c) J.A. Peters, J. Huskens and D.J. Raber, *Prog. NMR Spectroscopy*, **28**, 283 (1996). (d) J.H. Forsberg, in *Handbook on the Physics and Chemistry of Rare Earths*, Eds. K.A. Gschneidner and L.Eyring, Elsevier, Amsterdam, Vol. 23, Chapt.153, p. 1 (1996). (e) C.F.G.C. Geraldes and C. Luchinat, in *Metal Ions in Biological Systems*, Eds. A. Sigel and H. Sigel, Vol. 40, Chapt. 14, M. Dekker, New York, in press.
2. (a) P. Caravan, J. J. Ellison, T. J. McMurry and R. B. Lauffer, *Chem. Rev.* **99**, 229 (1999). (b) É. Tóth, L. Helm and A.E. Merbach, in *The Chemistry of Contrast Agents in Medical Magnetic Resonance Imaging*, Eds. A.E. Merbach and É. Tóth, Wiley, Chichester, p. 45 (2001).
3. (a) J.A. Peters, E. Zitha-Bovens, D.M. Corsi and C.F.G.C. Geraldes, in *The Chemistry of Contrast Agents in Medical Magnetic Resonance Imaging*, Eds. A.E. Merbach and É. Tóth, Wiley, Chichester, p. 315 (2001). (b) L. Frullano, J. Rohovec, J.A. Peters and C.F.G.C. Geraldes, *Top. Curr. Chem.*,221, 26 (2002).
4. D.J. Raber, in *Lanthanide Shift Reagents in Stereochemical Analysis*, VCH Publishers Inc., New York, Chapter 6 (1983).
5. R.M. Golding and M.P. Halton, *Aust. J. Chem.*, **25**, 2577 (1972).
6. B. Bleaney, *J. Magn. Reson.*, **8**, 91 (1972).
7. A.A. Pinkerton, M. Rossier and S. Spiliadis, *J. Magn. Reson.*, **64**, 420 (1985).
8. W. de W. Horrocks, Jr., J. P. Sipe III, *Science*, **177**, 994 (1972).
9. B.R. McGarvey, *J. Magn. Reson.*, **33**, 445 (1979).
10. J.M. Briggs, G.P. Moss, E.W. Randall and K.D. Sales, *J. Chem. Soc., Chem. Commun.*, 1180 **(1972)**.
11. C.N. Reilley, B.W. Good and J.F. Desreux, *Anal. Chem.*, **47**, 2110 (1975).
12. R. M. Golding and P. Pyykkö, *Mol. Phys.*, **26**, 1389 (1973).

13. J.A. Peters, *J. Magn. Reson.*, **68**, 240 (1986).
14. J. Reuben, *J. Magn. Reson.*, **50**, 233 (1982).
15. A.D. Sherry, M. Singh and C.F.G.C. Geraldes, *J. Magn, Reson.*, **66**, 551 (1986).
16. J. Ren and A.D. Sherry, *J. Magn. Reson.*, **B111**, 178 (1996).
17. J. Lisowski, J.L. Sessler, V. Lynch and T.D. Mody, *J. Am. Chem. Soc.*, **117**, 2273 (1995).
18. S. Spiliadis and A.A. Pinkerton, *J. Chem. Soc. Dalton Trans.*, 1815 **(1982)**.
19. C. Platas, F. Avecilla, A. de Blas, C.F.G.C. Geraldes, T. Rodriguez-Blas, H. Adams and J. Mahia, *Inorg. Chem.*, **38**, 3190 (1999).
20. J. Ren, S. Zhang, A.D. Sherry and C.F.G.C. Geraldes, *Inorg. Chim. Acta*, **339**, 273 (2002).
21. W. de W. Horrocks, Jr., *J. Magn. Reson.*, **26**, 333 (1977).
22. C.F.C.G. Geraldes, S. Zhang, C. Platas, T. Rodriguez-Blas, A. de Blas and A.D. Sherry, *J. Alloys and Compds*, **323/324**, 824 (2001).
23. N. Ouali, B. Bocquet, S. Rigault, P.-Y. Morgantini, J. Weber and C. Piguet, *Inorg. Chem.*,**41**,1436 (2002).
24. (a) S. Rigault and C. Piguet, *J. Am. Chem. Soc.*, **122**, 9304 (2000). (b) S. Rigault, C. Piguet and J.-C.G. Bünzli, *J. Chem. Soc., Dalton Trans.*, 2045 **(2000)**.
25. S. Rigault, C. Piguet, G. Bernardinelli and G. Hopfgartner, *J. Chem. Soc., Dalton Trans.*, 4587 **(2000)**.
26. C. Piguet and C.F.C.G. Geraldes, in *Handbook on the Physics and Chemistry of Rare Earths*, Eds. K.A. Gschneidner, L.Eyring, Elsevier, Amsterdam, in press.
27. C.F.G.C. Geraldes, S. Zhang, A.D. Sherry, *Inorg. Chim. Acta*, submitted.
28. A.A. Pinkerton and W. L. Earl, *J. Chem. Soc. Dalton Trans.*, 267 **(1978)**.
29. A.A. Pinkerton and D. Schwarzenbach, *J. Chem. Soc. Dalton Trans.*, 1470 **(1981)**.
30. S. Spiliadis, A.A. Pinkerton and D. Schwarzenbach, *J. Chem. Soc. Dalton Trans.*, 1809 **(1982)**.
31. S. Spiliadis, A.A. Pinkerton, D. Schwarzenbach, *Inorg. Chim. Acta*, **75**, 115 (1983).
32. S. Spiliadis and A.A. Pinkerton, *Inorg. Chim. Acta*, **75**, 125 (1983).
33. M.-R. Spirlet, J. Rebizant, J.F. Desreux , M.-F. Loncin, *Inorg. Chem.*, **23**, 359 (1984).
34. J.P. Dubost, J.M. Leger, M.H. Langlois, D. Meyer and M.C.R. Schaefer, *Academie Sci. Ser II Univers.*, **312**, 329 (1991).
35. C.A. Chang, L.C. Francesconi, M.F. Malley, K. Kumar, J.Z. Gougoutas, M.F. Tweedle, D.W. Lee and L.J. Wilson, *Inorg. Chem.*, **32**, 3501 (1993).
36. D. Parker, K. Pulukkody, F.C. Smith, A. Batsanov and J.A.K. Howard, *J. Chem. Soc. Dalton Trans.* 689 **(1994)**.
37. S.Aime, A.Barge, M.Botta, M.Fasano, J.D. Ayala and G.Bombieri, *Inorg.Chim.Acta,***246**, 423 (1996).
38. F. Benetollo, G. Bombieri, S. Aime and M. Botta, *Acta Cryst.*, **C55**, 353 (1999).
39. S. Aime, A. Barge, F. Benetollo, G. Bombieri, M.Botta and F.Uggeri, *Inorg. Chem.*, **36**, 4287 (1997).
40. J.F. Desreux, *Inorg. Chem.*, **19**, 1319 (1980).
41. S. Aime, M. Botta and G. Ermondi, *Inorg. Chem.*, **31**, 4291 (1992).
42. S. Aime, L. Barbero, M. Botta and G. Ermondi, *J. Chem. Soc. Dalton Trans.*, 225 **(1992)**.
43. S. Hoeft and K. Roth, *Chem. Ber.*, **126**, 869 (1993).

44. M.P.M. Marques, C.F.G.C. Geraldes, A.D. Sherry, A.E. Merbach, H. Powell, D. Pubanz, S. Aime and M. Botta, *J. Alloys and Compds*, **225**, 303 (1995).
45. S. Aime, M. Botta, M. Fasano, M.P.M. Marques, C.F.G.C. Geraldes, D. Pubanz and A.E. Merbach, *Inorg. Chem.* **36**, 2059 (1997).
46. V. Jacques and J.F. Desreux, *Inorg. Chem.*, **33**, 4048 (1994).
47. J.R. Morrow, S. Amin, C.H. Lake and M.R. Churchill, *Inorg. Chem.*, , **32**, 4566 (1993).
48. S. Amin, J.R. Morrow, C.H. Lake and M.R. Churchill, *Angew. Chem. Int. Ed. Engl.*, **33**, 773 (1994).
49. A.. Bianchi, L. Calabi, C. Giorgi, P. Losi, P. Mariani, P. Paoli, P. Rossi, B. Valtancoli and M. Virtuani, *J. Chem. Soc. Dalton Trans.*, 697 (**2000**).
50. S. Aime, A. Barge, J.I. Bruce, M. Botta, J.A.K. Howard, J.M. Moloney, D. Parker, A.S. de Sousa and M. Woods, *J. Am. Chem. Soc.*, **121**, 5762 (1999).
51. J.H. Forsberg, R.M. Delaney, Q. Zhao, G. Harakas and R. Chandran, *Inorg. Chem.*, **34**, 3705 (1995).
52. E.F. Paulus and P. Juretschke, J. Lang, *3 Jahrestag der Deutschen Gesellschaft für Kristallographie*, Darmstadt, Germany (1995).
53. C.F.G.C. Geraldes, A.D. Sherry and G.E. Kiefer, *J. Magn. Reson.*, **97**, 290 (1992).
54. A.D. Sherry, J. Ren, J. Huskens, E. Brücher, É. Tóth, C.F.G.C. Geraldes, M.M.C.A. Castro and W.P. Cacheris, *Inorg. Chem.*, **35**, 4604 (1996).
55. K. Kasuga, M. Tutui, R.C. Peterson, T. Tatsumi, N. VanOpdenbosch, G. Pepe and E.F. Meyer Jr., *J. Am. Chem. Soc.*, **102**, 4835 (1980).
56. M. Moussavi, A. De Cian, J. Fischer and R. Weiss, *Inorg. Chem.*, **27**, 1287 (1988).
57. H. Konami, M. Hatano and A. Tajiri, *Chem. Phys. Lett.*, **160**, 163 (1989).

